# Emerging Insights into Targeted Therapy-Tolerant Persister Cells in Cancer

**DOI:** 10.3390/cancers13112666

**Published:** 2021-05-28

**Authors:** Heidie Frisco Cabanos, Aaron N. Hata

**Affiliations:** 1 Massachusetts General Hospital Cancer Center, Charlestown, MA 02129, USA; hfcabanos@up.edu.ph; 2 Department of Medicine, Harvard Medical School, Boston, MA 02115, USA

**Keywords:** drug-tolerant persisters, targeted therapy, acquired drug resistance

## Abstract

**Simple Summary:**

Acquired resistance to molecularly targeted therapies remains a major challenge in the treatment of cancer. It has been hypothesized that drug-tolerant (or “persister”) cells without *bona fide* resistance mechanisms may survive initial drug treatment and undergo further evolution over time to acquire resistance mechanisms leading to cancer relapse. In this review, we will discuss insights into mechanisms and vulnerabilities of these cells revealed by recent in vitro, in vivo, and clinical studies.

**Abstract:**

Drug resistance is perhaps the greatest challenge in improving outcomes for cancer patients undergoing treatment with targeted therapies. It is becoming clear that “persisters,” a subpopulation of drug-tolerant cells found in cancer populations, play a critical role in the development of drug resistance. Persisters are able to maintain viability under therapy but are typically slow cycling or dormant. These cells do not harbor classic drug resistance driver alterations, and their partial resistance phenotype is transient and reversible upon removal of the drug. In the clinic, the persister state most closely corresponds to minimal residual disease from which relapse can occur if treatment is discontinued or if acquired drug resistance develops in response to continuous therapy. Thus, eliminating persister cells will be crucial to improve outcomes for cancer patients. Using lung cancer targeted therapies as a primary paradigm, this review will give an overview of the characteristics of drug-tolerant persister cells, mechanisms associated with drug tolerance, and potential therapeutic opportunities to target this persister cell population in tumors.

## 1. Introduction

Over the past decades, we have seen the development and evolution of molecularly targeted therapies for treating cancer. In particular, advances in our understanding of somatic genetic alterations or “driver” mutations in oncogenic kinases have given rise to effective therapies that target specific oncogenic signaling pathways, which often leads to profound clinical responses. Examples of these targeted cancer therapeutics that have markedly improved patient survival are tyrosine kinase inhibitors (TKIs) that block or suppress the activity of oncogenic driver receptor tyrosine kinases (RTKs), such as epidermal growth factor receptor (EGFR) and echinoderm microtubule-associated protein-like 4 gene, fused to the anaplastic lymphoma kinase (EML4-ALK) in non-small cell lung cancer (NSCLC), as well as mitogen-activated protein kinase (MAPK) pathway-directed agents against mutant B-Raf (BRAF) in melanoma and other solid tumors [1,2,3,4,5,6,7]. Unfortunately, the success of these therapies is limited by drug resistance that ultimately renders the treatment ineffective.

**Figure 1:**
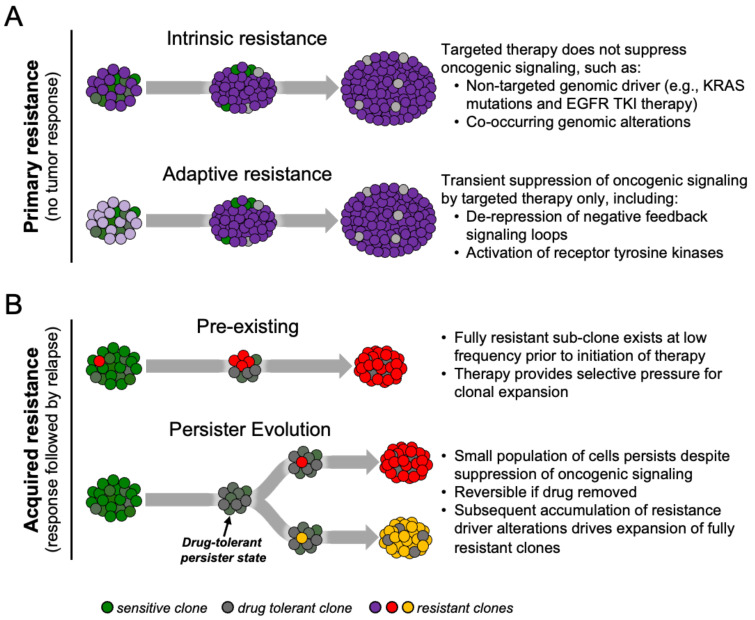
Resistance to targeted cancer therapies. Drug resistance can occur at the time of initial therapy (primary resistance) or develop after initial therapy response (acquired resistance). (**A**) Primary resistance may be due to intrinsic resistance resulting from ineffective targeting of the oncogenic driver such that oncogenic signaling is not suppressed (for instance, activating KRAS mutations confer resistance to EGFR targeted therapy). Alternatively, in adaptive resistance, cells rapidly rewire oncogenic signaling after initial suppression such that therapy does not induce death in the bulk of tumor cells. (**B**) In acquired drug resistance, disease relapse after initial therapy response may be driven by Darwinian selection of resistant clones that exist before treatment and expand under the selective pressure of therapy (for instance, rare T790M+ clones in some EGFR-mutant lung cancers), or evolution of drug-tolerant “persister” cells that acquire resistance mechanisms during the course of therapy.

Drug resistance to targeted therapies in cancer can be classified as primary or acquired (Figure 1A) [8,9]. Primary resistance refers to the ability of cancers to evade initial therapy. Clinically, this refers to non-responders that exhibit lack of tumor shrinkage after initiation of treatment and is thought to be due to intrinsic resistance due to a pre-existing genetic alteration or predominant resistant cell-state within the treatment-naïve tumor, or the ability of cells to rapidly adapt to therapy (so-called adaptive resistance).

On the other hand, acquired drug resistance develops after prolonged treatment of tumors that initially responded to therapy. Mechanisms of acquired resistance to targeted therapies, especially those of kinase inhibitors, have been described over the past several years. These include genetic drivers of drug resistance such as secondary mutations in the targeted oncogene, activation of bypass signaling pathways, and lineage transformation [10]. In the first mechanism, tumor cells can have pre-existing or develop genetic alterations that confer drug resistance to the specific targeted inhibitor by increasing catalytic activity or impairing binding of the drug to the target oncogene. For example, in EGFR-mutant lung cancers, the point mutation leading to a substitution of methionine for threonine at amino acid position 790 in exon 20 of EGFR, known as the EGFR T790M gatekeeper mutation, is a leading cause of resistance to first- and second-generation EGFR inhibitors [11,12]. The T790M mutation impairs the binding of the TKI to the tyrosine kinase due to steric hindrance of the bulkier methionine side chain in the ATP-kinase-binding pocket as well as increasing the affinity of mutant EGFR for ATP [13]. In the second mechanism, downstream signaling pathways are reactivated through mechanisms independent of the original drug target. Redundancy and feedback mechanisms in the signaling pathways allow the tumor cells to confer “bypasses” in oncogenic signaling, resulting in suppression of the targeted pathway which leads to resistance. For example, MET activation, by either gene amplification or ligand stimulation, accounts for 5–10% of lung cancer resistance to EGFR TKIs [12,14]. In experimental models of MET-driven acquired resistance, MET reactivates both PI3K/AKT and MEK/ERK signaling despite EGFR inhibition [14,15]. A study by Wilson and colleagues showed that many growth factors can reduce sensitivity of many different oncogene-addicted cancer models to TKIs, highlighting the redundancy of kinase signaling in cancer cells [16]. A classic example of the third mechanism of acquired resistance—lineage transformation—is the histologic transformation of lung adenocarcinoma subtype to small cell carcinoma subtype, which accounts for 5–15% of EGFR-mutant lung cancer patients with acquired resistance to EGFR TKIs [12,17]. Although the molecular mechanisms underlying this lineage transformation still remain to be elucidated, studies suggest that certain genomic features (e.g., loss of both TP53 and RB1) are required for this phenotypic transformation to occur [18,19].

Although there are many studies elucidating specific mechanisms of acquired drug resistance, less is known about how resistant clones emerge and evolve during therapy. Traditionally, mechanisms of acquired resistance have been viewed in two ways—pre-existing or de novo evolution (Figure 1B). Given the potential clonal heterogeneity that exists within a tumor, rare subclones may harbor mechanisms of acquired drug resistance even before the initiation of therapy. These resistant cells expand during the period of initial response to targeted therapy, eventually driving tumor relapse due to “acquisition” of the drug resistance mechanism [15,20,21,22]. Alternatively, cells may survive initial drug treatment by epigenetic adaptations that enable a drug-tolerant, slow-cycling phenotype that precedes the emergence of permanent drug resistance [23,24]. These tumor subpopulations can evade strong selective drug pressure by entering this drug-tolerant “persister” state which can then serve as a cellular reservoir from which clones that evolve bona fide drug-resistance mechanisms can emerge [25,26]. In the clinic this most closely corresponds to minimum residual disease (MRD), or the point of maximal tumor shrinkage prior to eventual tumor progression [8,27,28].

In this review, we provide an overview of the characteristics of drug-tolerant persister cells in cancer, how they arise, and the mechanisms these cells utilize to evade targeted therapy. To note, acquired resistance or tolerance to chemotherapies as well as cancer immunotherapies has also been reported [29], but this review specifically focuses on targeted therapies for solid malignancies.

## 2. Origins and Characteristics of Persisters

### 2.1. Bacterial Persisters

**Figure 2:**
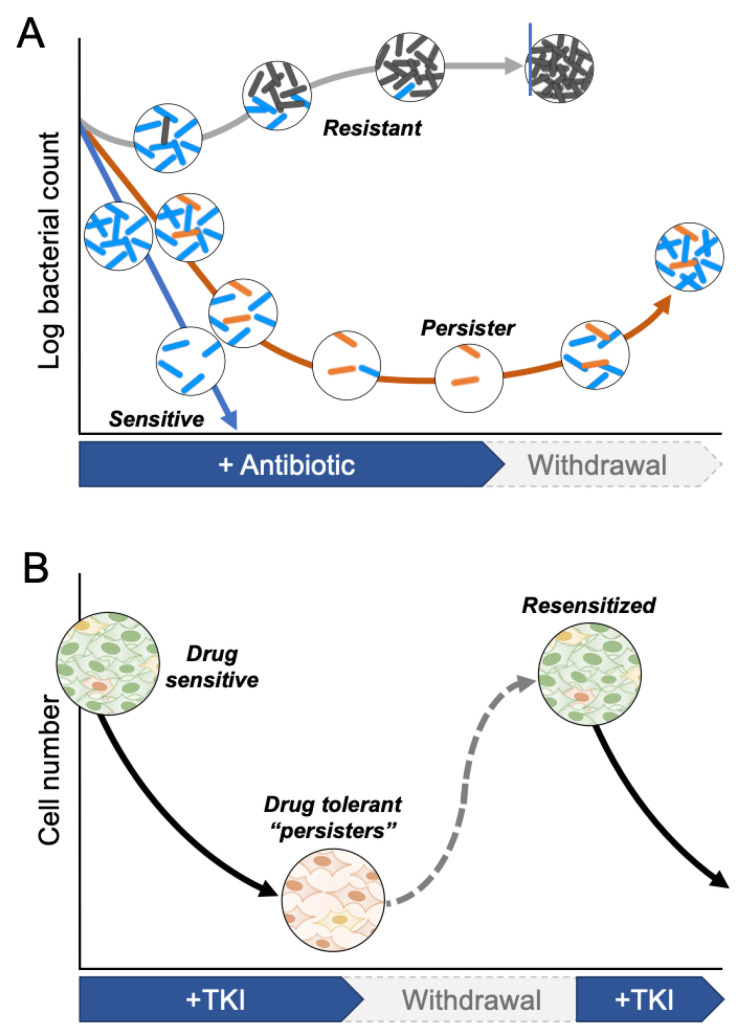
Conceptual similarities between bacterial and cancer persisters. (**A**) Survival curves depict bacterial state trajectories during exposure to antibiotics. Sensitive bacterial populations (blue) are eliminated by antibiotic treatment, whereas resistant bacteria (dark gray) continue to proliferate in the presence of antibiotic. Persister bacteria (orange) can survive during antibiotic exposure, but upon drug withdrawal, repopulate rapidly, proliferating sensitive bacterial populations. (**B**) Prototypical survival curve illustrating the emergence and reversibility of drug-tolerant cancer cells from a primarily drug-sensitive population. Treatment of heterogenous tumor cell populations with targeted therapy eliminates sensitive clones, revealing a subpopulation of so-called “drug-tolerant persister cells” (DTPs). A hallmark of the persister state is reversibility upon withdrawal of drug, resulting in re-establishment of a mixed population of sensitive and drug-tolerant cells.

The concept of a drug-tolerant persister state has its origins in the field of microbiology in the 1940s, shortly after the discovery of antibiotics [30,31,32]. In 1942, Gladys Hobby observed that while penicillin was capable of killing the vast majority of cultured streptococcal cells, ~1% remained intact [33]. The surviving bacterial cells were later characterized and named “bacterial persisters” in 1944 by Joseph Bigger, who hypothesized that their survival resulted from slow cell cycle rates rather than innate heritable resistance mechanisms [34]. More recently, live imaging experiments have confirmed that these bacterial cells that survived antibiotic treatment were indeed not proliferating before the addition of antibiotics [30]. Moreover, upon drug withdrawal, these drug-tolerant slow-cycling bacteria that survived antibiotic treatment can revert to a proliferative state and re-establish a drug sensitive population, demonstrating that bacterial drug persistence is transient and mediated by phenotypic plasticity rather than genetic alterations (Figure 2A) [31,32].

### 2.2. Persisters in Cancer

**Figure 3:**
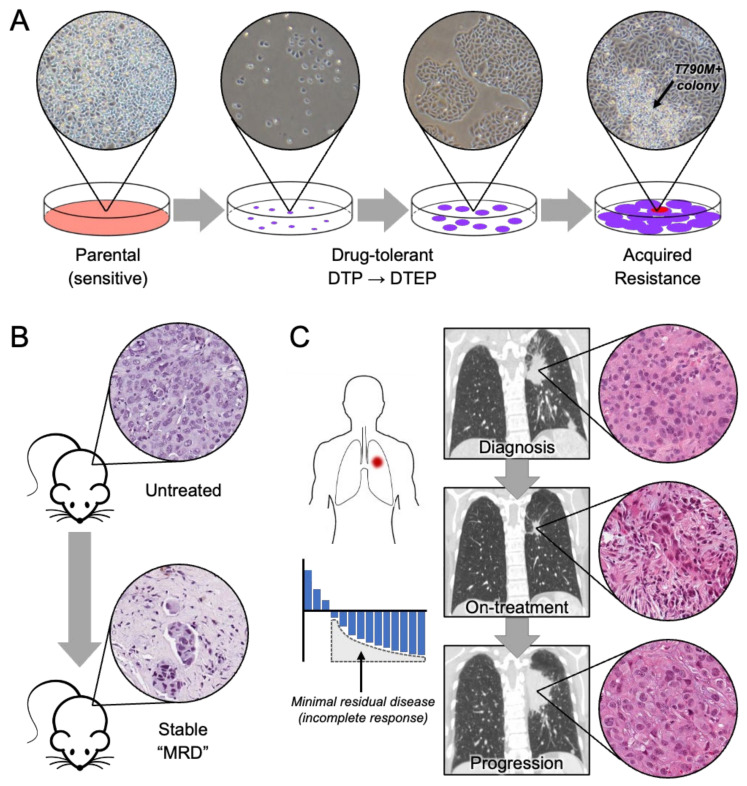
Studying persisters in cancer. (**A**) Modeling persisters using in vitro cell culture systems. Persisters are modeled by a sub-population of cells that remain viable in culture upon extended drug treatment after the majority of sensitive parental cells are eliminated. PC9 EGFR-mutant lung cancer cells are shown before (left) and during gefitinib treatment. Over time, the population of drug-tolerant persisters (middle images) gives rise to a fully resistant T790M+ clone (right image). (**B**) Modeling persisters in vivo using mouse xenograft tumors. Drug treatment leads to tumor regression followed by a period of stable “minimal residual disease” (MRD) in which a small population of cancer cells exist within tumors that are no longer regressing. Shown are H&E images of EGFR-mutant lung cancer patient-derived xenograft tumors before or after treatment with osimertinib, revealing small residual clusters of surviving tumor cells. (**C**) Studying persisters in the clinic. As illustrated by a typical waterfall plot of best tumor response, most patients do not experience complete responses to targeted therapies. Residual disease lesions harbor surviving cancer cells but may remain stable for months or even years. Sequential pre/on-treatment and progression biopsies enable study of persister phenotypes and the evolutionary trajectories of resistant cancer cells. H&E images depict sequential biopsies from an EGFR lung cancer patient treated with EGFR targeted therapy in a clinical trial. In the on-treatment biopsy, small clusters of tumors cells are surrounded by extensive fibroblastic stroma.

The notion of persisters was extended to the cancer field in 2010 by Settleman and colleagues to describe cancer cells treated with targeted therapies [23]. Using the well-described human NSCLC cell line PC9, which harbors an oncogenic EGFR exon 19 deletion, they observed that a small fraction constituting ~0.3% of the original population survived treatment with a lethal dose of the EGFR inhibitor erlotinib. They termed this subpopulation of slowly cycling quiescent cells drug-tolerant persisters (DTPs). Over time, a fraction of DTPs reinitiated proliferation in the presence of erlotinib, giving rise to a second population of cells labeled drug-tolerant expanded persisters (DTEPs). Similar to bacterial persisters, DTEPs removed from the drug reverted to a drug-sensitive state. The authors also tested other cell lines, including other lung cancer, melanoma, colorectal cancer, and breast cancer cell lines, and observed the selection of a similar subpopulation of drug-tolerant persister cells after exposure to various drugs (cisplatin, EGFR inhibitors, RAF inhibitor, and MET inhibitor). As a result, a subpopulation of cancer cells that is drug-tolerant cancer is widely present within tumor-derived cell lines and emerges upon treatment with targeted therapies (Figure 2B and Figure 3A).

Subsequently, work by our group and others showed that clinical mechanisms of acquired resistance can occur by evolution of drug-tolerant persister cells during long term drug exposure [25,26]. In our study, we treated multiple small pools of PC9 cells with the EGFR inhibitor gefitinib until resistant clones emerged [25]. This resulted in two treatment outcomes: a minority of pools (5–10%) developed rapid resistance due to acquisition of the EGFR^T790M^ resistance mutation as early as 2 weeks after initiating drug treatment (early-resistant clones), while most pools initially underwent a dramatic initial drug response followed by slow expansion of a small subpopulation of surviving, drug-tolerant cells, and finally (up to a year later) the development of full drug resistance (late-resistant clones). Lineage tracing with DNA barcoding confirmed that the early-resistant clones were derived from rare pre-existing EGFR^T790M^-positive cells that existed in the parental PC9 cell line. On the other hand, PC9 single-cell cultures that did not harbor pre-existing EGFR^T790M^ cells eventually gave rise to EGFR^T790M^ or other genomic mechanisms of resistance when treated with EGFR inhibitor continuously for extended periods of time, demonstrating de novo evolution of drug resistance mechanisms from persisters. These findings were corroborated with transcriptional profiling showing that the late-resistant clones retained features of the drug-tolerant state, while the early-resistant clones showed transcriptional profiles more similar to that of the parental cells. Similar conclusions were reached by Altschuler and colleagues, who derived a panel of erlotinib-resistant cell lines from single PC9 drug-tolerant cells. Collectively, these resistant cells recapitulated multiple mechanisms of acquired resistance observed in the clinic, including EGFR^T790M^ and MET amplification [26]. Thus, these studies suggest that the drug-tolerant persister state may comprise a reservoir of cells from which heterogeneous drug-resistance mechanisms can evolve.

Following the initial description by Settleman and colleagues, similar drug-tolerant persisters have been reported in many other cancer models, including lung cancer [35,36,37,38,39,40], melanoma [39,40,41], breast cancer [39,40,42], colon cancer [39], gastric cancer [43], ovarian cancer [40], leukemia [44], and glioblastoma [45]. In vivo models have shown similar drug-tolerant slow-cycling persister cells. Drug-tolerant quiescent residual cells in basal cell carcinoma (BCC) can cause relapse after treatment is stopped [46,47]. A slow-cycling starvation-like state in melanoma may be a required intermediate phase in the development of minimal residual disease [48].

In contrast to bacterial persistence, the terminology of “persisters” in cancer is often more loosely applied. In microbiology, resistance is defined as the inherent ability of bacteria to grow at high concentrations of antibiotics and typically results from genetic mutations [49]. To differentiate modes of survival from resistance and drug susceptibility, the expressions “tolerance” and “persistence” are used in bacteria [50]. Tolerance in bacteria refers to the ability to survive transient exposure of a drug, regardless of whether it is inherited or not [51]. Persistence in bacteria is the ability of a sub-clonal population of cells to survive treatment [51]. In cancer, the term “persister” has no clear biological definition and the term is used interchangeably with other terms such as tolerant [23,25,40], dormant [52], or quiescent [53] to refer to sub-populations of cancer cells that survive prolonged drug treatment [54]. In general, these cell populations or states exist in between sensitive and resistant states, a period during drug treatment in which the cells are alive but not fully proliferative nor resistant. In contrast to drug resistance, drug-tolerant persister cells are “tolerant” to the drug in the sense that the drug does not induce cell death, but still exerts a suppressive effect. In the clinic, this corresponds to the period of time after maximal tumor regression but before quantifiable tumor growth, despite continued therapy (Figure 3C).

Although their distinction and how they relate to the progression of completely drug-resistant tumors is incompletely defined, persisters in cancer share several common themes. The first theme is that persister cells make up a small population of cells that remains viable despite drug exposure. A characteristic feature of drug-tolerant cells is evasion of drug-induced apoptosis [25]. The second theme is that persisters are slow cycling, or alternatively termed dormant or quiescent, with little-to-no population growth [23,55]. Entering a quiescent or dormant state characterized by slow cycling may facilitate changes in metabolism and other survival mechanisms which allow cancer cells to survive drug exposure over long periods of time [52,54]. Interestingly, this dormant state can share features with cellular senescence. For example, EGFR-mutant lung cancer cells that survive treatment with combined EGFR and MEK inhibitors express hallmarks of senescence such as an increase in senescence-associated gene expression signature, senescence-associated b-galactosidase activity (SA-b-Gal), increased secretion of several classical SASP (senescence-associated secretory phenotype) factors, robust induction of p27kip, and punctate H3K9Me3-positive nuclear foci [56]. The third theme is that mechanisms of cancer cell persistence are non-genetic, supported by the observation that the drug-tolerant state is transient and reversible upon removal of the drug, reminiscent of bacterial persisters. Unlike treatment-induced senescence that occurs as a result of DNA-damaging chemotherapeutic agents, cancer cells seem to exhibit the reversible drug-tolerant state most commonly upon treatment with targeted therapy [57]. This reversibility of drug-tolerant persister states suggests underlying epigenetic mechanisms. In some cases, epigenetic mechanisms of drug tolerance coincide with expression of stem cell markers, suggesting that persisters may share features of cancer stem cells [23,24,58]. For an excellent review on cancer stem cells and anticancer drug resistance, we refer the reader to an excellent review by Shibue and Weinberg [59].

### 2.3. Origin of Drug-Tolerant Persister Cells

Given that persisters can be a source of acquired drug resistance, understanding how persister cells arise may help guide therapeutic strategies to target them and prevent or delay the development of drug resistance. Some studies indicate that drug-tolerant persister populations or cancer cells that are primed to persist exist even prior to treatment [40,43,60,61]. To formally address whether the onset of dormancy after drug treatment is a predetermined or a stochastic process, Kurppa et al. used DNA barcoding on PC9 cells treated with EGFR inhibitor alone, MEK inhibitor alone, or both [56]. The results revealed a large fraction of shared barcodes within the cells treated with EGFR inhibitor alone, consistent with selection of pre-existing persister clones. Schaffer et al. showed that human BRAF mutant melanoma cells exhibit transcriptional heterogeneity at the single-cell level, defining which cells will eventually resist drug therapy [61]. This stochastic variability led to a small subpopulation of cells transiently displaying simultaneous high expression of multiple resistance genes such as WNT5, AXL, EGFR, PDGFRB and JUN concurrently, increasing the probability of surviving vemurafenib treatment [61]. Hangaeur et al. found that pretreatment of HER2-amplified breast cancer line BT474 parental cells with the GPX4 inhibitor RSL3 reduces the amount of persister cells that remain after subsequent treatment with the HER2 inhibitor lapatinib, suggesting that GPX4-sensitive cells pre-exist [40]. Both these findings are consistent with previous reports suggesting that persister cells may pre-exist before drug treatment [43,60]. Collectively, these studies support a model wherein a pre-existing sub-population of drug-tolerant tumor cells is enriched upon drug treatment, akin to the principle of non-genetic Darwinian selection [60,62,63]. In other words, pre-existing heterogeneity provides the basis for selection rather than plasticity.

Meanwhile, other studies suggest that some persister phenotypes arise via plasticity induced by drug treatment [56,64,65]. In the same DNA barcoding study by Kurppa et al. described above, the authors observed that after treatment with the combination of EGFR and MEK inhibitors, the vast majority of barcodes were unique, suggesting that the ability of cells to enter dormancy following dual inhibition of EGFR and MEK is predominately driven by a stochastic process [56]. Risom et al. showed that a MEK and PI3K/mTOR inhibitor-driven drug-tolerant persistent state in breast cancer arises through distinct cell-state transitions which involves dynamic remodeling of open chromatin architecture [66], in contrast to simple Darwinian selection of pre-existing subpopulations. Lee et al. found that transient STAT3 activation mediated by IL-6 and FGFR in response to oncogenic kinase inhibition significantly contributes to survival of several different oncogene-addicted cancer cell lines [67]. The authors hypothesized that a stochastically driven competition between the rates of engagement of the apoptotic machinery and the cell-protective feedback signaling pathway ultimately determines the fates of cells, given the relatively slow kinetics associated with STAT3 feedback activation and the relatively rapid suppression of oncogenic survival signals observed following drug treatment. On the other hand, it is also conceivable that, prior to treatment, a subpopulation of cancer cells is primed to quickly engage the STAT3 feedback system during drug treatment.

It is important to note that pre-existing and induced persister states are not mutually exclusive. Stochastic gene expression across the population could contribute to phenotypic heterogeneity that at the time of treatment initiation determines which cells will survive long enough to reprogram a more stable resistance phenotype [68]. The study by Schaffer et al. discussed above revealed that the initial selection of a sub-population of cells based on pre-existing transcriptional variability and concurrent expression of multiple resistance genes was subsequently followed by drug-induced epigenetic reprogramming, converting the transient transcriptional state to a stably resistant state. In this case, reprogramming involved loss of SOX10-mediated differentiation and was followed by activation of new signaling pathways partially mediated by JUN and/or AP-1 and TEAD transcription factors [61].

## 3. Drug-Tolerant Persister Cells: Mechanisms of Drug Tolerance and Therapeutic Vulnerabilities

Our current understanding of the different mechanisms that drug-tolerant persister cells utilize to survive drug exposure focuses primarily on in vitro studies utilizing patient-derived cell lines or in vivo xenograft models (Figure 3A,B), where drug titration may allow for selection of drug-tolerant cells. In this section, we will discuss past and recent studies investigating the mechanistic basis of drug tolerance (see Table 1 for summary of the mechanisms of drug tolerance).

### 3.1. Epigenetic Reprogramming/Plasticity

Epigenetic reprogramming may be of central importance when drug-resistant states are functionally heterogeneous, evolving, and transient [69], in contrast to more stable drug resistance resulting from genetic alterations. Tumors can display a constantly evolving epigenetic landscape that involves altered DNA promoter regions shifts, deregulated histone protein acetylation or methylation, or dysregulated expression of repetitive elements [70]. A number of studies have now provided convincing evidence that cancer cells can become refractory to therapy by acquiring a drug-tolerant state through epigenetic mechanisms that can conversely be reversed by epigenetic reprogramming.

The initial description of EGFR-mutant NSCLC drug-tolerant persister cells by Sharma et al. revealed that these cells have a globally repressed chromatin state [23]. One gene overexpressed in the persister population was KDM5A (also known as JARID1A) which encodes a histone demethylase that removes methyl groups on histone H3K4, a mark of active transcription. Interestingly, acetylation of H3K14 was also observed to be decreased in drug-tolerant persisters, suggesting interactions between multiple epigenetic regulators. Indeed, knockdown of KDM5A or treatment with histone deacetylase (HDAC) inhibitors reduced the survival of persister cells. Subsequent studies by this same group extended these findings to show that the repressive chromatin landscape of EGFR-mutant NSCLC persisters results from histone modifications orchestrated by the action of multiple histone methyltransferases and HDACs including SETDB1, G9a, EZH2, HP1, ATRX, and class I HDACs [24]. Genetic knockdown of genes that promote H3K9me3-mediated heterochromatin formation (such as ATRX, SETDB1, G9a, and several class I HDACs) decreased persister survival, indicating that H3K9 methylation–dependent heterochromatin formation is required to establish the drug-tolerant state.

The crucial role of histone demethylases in establishing a slow-dividing and drug-tolerant or resistant state has now been corroborated by a number of studies using a variety of different experimental models. Upregulation of histone demethylases (KDMs) leads to changes in the methylation of histone3 lysine (H3K) residues, as well as deregulation of a variety of cellular functions [39]. KDM2 [71], KDM3 [45], KDM5 [23,39,45,60,72], and KDM6 [45] have been found to be upregulated in different tumor models of drug tolerance and resistance to various drugs. In melanoma, persister cells express elevated levels of KDM5 leading to slowing of cell cycle progression [60,72]. The exposure of glioblastoma stem cells to dasatinib, a kinase inhibitor, induced a transition to a slow-cycling persistent state marked by repressive chromatin redistribution and KDM6 dependence [45].

Changes in global chromatin architecture can lead to remodeling of enhancers and super-enhancers that control transcriptional programs, leading to a state of transcriptional dependency or addiction [73]. Epigenetic readers such as bromodomain and extraterminal (BET) proteins play a major role in mediating these changes to establish drug tolerance in some contexts. Several studies have revealed that BET proteins facilitate chromatin remodeling and transcriptional adaptation necessary for establishing the DTP state in response to MEK and PI3K inhibitors in breast cancer models, which can be prevented by the BET inhibitor JQ1 [66,74]. BET proteins such as BRD4 have been shown to be targeted to specific areas of the genome by interaction with other DNA-binding proteins such as YAP/TAZ to enforce transcriptional addiction in cancer cells [75]. YAP/TAZ have been shown to play a role in establishing the DTP state in EGFR-mutant NSCLC cells treated with combination EGFR and MEK inhibitors by transcriptional reprogramming of apoptotic pathways [56]; however, whether BRD4 or other BET proteins play a role in this process remains to be elucidated. Other studies have demonstrated that targeting RNA polymerase II-mediated transcription with the CDK7/12 inhibitor THZ1 [76] synergizes with various targeted therapies to suppress drug-tolerant persister cells [77], further supporting a role for transcriptional adaptation in the establishment of the DTP state.

Epigenetic reprogramming of drug-tolerant persisters can also lead to altered regulation of cell fate and lineage drivers. For instance, epithelial-to-mesenchymal transition (EMT) is a phenotype switching event induced by targeted therapy in various tumors that has been linked to drug tolerance and/or resistance [78,79]. In melanoma, tumor cells treated with targeted therapy underwent a lineage transition to an undifferentiated mesenchymal cell state leading to drug tolerance and eventually resistance [48,80,81]. In basal cell carcinoma (BCC), comparison of genome-wide chromatin accessibility profiles in drug-naive and residual tumor cells after hedgehog pathway inhibition revealed that regions of increased chromatin accessibility in residual cells were enriched for transcription factor-binding motifs, including SOX family transcription factors that regulate stem and progenitor cell fate [46]. These changes coincided with a switch in stem cell identity driven by remodeling of super-enhancer regions that control main lineage-specific transcription factors, including IFE-specific KLF5 and bulge-specific LHX2. In melanoma, core regulators of neural crest lineage, MITF and SOX10, regulate the emergence of distinct cell states associated with drug tolerance establishment after treatment [61,82,83,84]. Beyond melanoma, SOX10 has been shown to mediate mammary duct cell state interconversion and promote de-differentiation to a more stem/progenitor state characterized by a neural crest-like phenotype in breast tumors [85]. Other master regulators of core developmental programs, such as enhancer of zeste homologue 2 (EZH2), which is the catalytic subunit of the Polycomb repressive complex 2 (PRC2), are emerging as key players driving the epigenetic changes driving drug tolerance. In lung and breast cancer, including EGFR-mutant NSCLC cells treated with EGFR inhibitors, drug-tolerant persisters show an increase in methylation on K116 of PRC member Jarid2, which helps stabilize and recruit PRC2 to chromatin, according to proteome-wide comparisons of lysine methylation patterns [86]. In addition to methylating histone H3K27, EZH2 can also methylate G9a at K185, which enhances the ability for G9a to recruit repressive complexes to chromatin. Thus, EZH2, either directly through remodeling of histones or indirectly through its ability to regulate other chromatin remodeling enzymes, may play a central role in orchestrating the various epigenetic changes that ultimately enable the establishment of drug-tolerant persister precursors.

**Table 1:** Studies on drug-tolerant persister cells to elucidate mechanisms of persistence using in vitro and in vivo experimental cancer models.

Tumor Type	Target Oncogene	Targeted Therapy	Model System	Mechanism of Drug Tolerance	Susceptibility	Reference
**Epigenetic programming/plasticity**						
NSCLC	EGFR	Gefitinib	PC9	Repressed chromatin state (KDM5 upregulation; H3K9 methylation-dependent LINE-1 elements)	HDAC (trichostatin)	[23,24]
		Erlotinib	PC9	Cell fate and lineage plasticity (increased methylation on K116 of Jarid2, with attendant stabilization and recruitment to chromatin of the PRC2 complex)	EZH2 (EPZ-6438)	[86]
	EGFR, ALK	Erlotinib, crizotinib	PC9 and xenograft, H3122	Dynamic transcriptional responses—remodeling of enhancers	CDK7/12 (THZ1)	[77]
Breast	HER2, EGFR, PI3K	Lapatanib	SKBR3, EVSA-T	Repressed chromatin state (KDM5 upregulation)	KDM5 (CPI-455)	[39]
	HER2, EGFR	Lapatinib	SKBR	Cell fate and lineage plasticity (increased methylation on K116 of Jarid2, stabilization, and recruitment to chromatin of the PRC2 complex)	EZH2 (EPZ-6438)	[86]
	MEK	Trametinib	SUM-159PT, HCC1806; xenografts	Transcriptional adaptation	BET (JQ1 and I-BET151)	[74]
	MEK and PI3K/mTOR	Trametinib and AZD6244; BEZ235 and PI103	HCC1143, SUM149PT	Transcriptional adaptation	BET inhibitor (JQ1)	[66]
Melanoma	BRAF, c-Raf-1	AZ628	Hs888, M14	Repressed chromatin state (KDM5 upregulation)	KDM5 (CPI-455)	[39]
	BRAF	Vemurafenib, bortezomib	WM3734 and xenograft	Repressed chromatin state (H3K4-demethylase JARID1B/KDM5B/PLU-1)	Mitochondrial enzyme (oligomycin, Bz-423, rotenone and phenformin, antimycin A, oligomycin)	[60]
	BRAF, MEK	Dabrafenib, trametinib	PDX	Cell fate and lineage plasticity (EMT)	RXR (HX531)	[48]
	RAF, MEK, ERK	Vemurafenib, selumetinib, ERK inhibitor	A375, WM266.4	Cell fate and lineage plasticity (high MITF expression)	HDAC (panobinostat, vorinostat, or entinostat); forskolin and IBMX (FSK/I)	[82]
Glioblastoma	RTK	Dasatinib	GSC6 and GSC8	Adaptive chromatin remodeling (KDM3, KDM6 upregulation)	KDM (GSKJ4)	[45]
Colon cancer	BRAF, c-Raf-1	Vemurafenib	Colo205	Repressed chromatin state—KDM5 upregulation	KDM5 (CPI-455)	[39]
Bladder carcinoma	FGFR	BGJ398	RT112 and xenograft	Dynamic transcriptional response (remodeling of enhancers)	CDK7/12 (THZ1)	[77]
Basal cell carcinoma	Hedgehog (Smoothened)	Vismodegib	Ptch1–Trp53 mouse model	Cell identity switch (Wnt pathway activation and reprogramming of super-enhancers)	Wnt (function-blocking anti-LRP6 antibody)	[46]
**Activation of bypass and alternative signaling pathways**						
NSCLC	EGFR	Erlotinib	HCC827 and xenograft	AXL kinase signaling	AXL (MP-470 or XL-880)	[87]
			HCC827, HCC4006, and xenograft	Notch3-dependent β-catenin signaling	β-catenin (XAV939, ICG-001)	[88]
			H1650 and xenograft	TGF-B-IL-6-STAT3 axis	Innate immune (12-O-tetradecanoyl- and LPS)	[89]
			HCC827, PDX	NF-kB signaling	NF-kB (PBS-1086)	[90]
			HCC827 and HCC4006	YAP/FOXM1 axis	CDK (dinaciclib, alvocidib), SAC components (volasertib, ON-01910, ispinesib, SB743921, AMG-900, alisertib)	[91]
		Osimertinib	PC9	AXL kinase signaling	AXL (NPS1034)	[92]
		EGF816	PDCL	FGFR signaling	FGFR (BGJ398)	[93]
		Osimertinib, rociletinib	PC9, HCC827, HCC4006, and NCI-H1975	AURKA signaling	AURKA (MLN8237)	[94]
	EGFR, MEK	Selumetinib	PC9, HCC827, HCC2935	FGF-JAK kinase-Stat3 signaling	JAK (ponatinib/ruxolitinib) FGF (PD173074) inhibitors	[67]
		Osimertinib, trametinib	PC9, HCC827, HCC4006; xenograft	YAP/TEAD/SLUG signaling	YAP (XAV939)	[56]
Melanoma	BRAF, MAPK	Vemurafenib, trametinib	PDX	AXL signaling	AXL antibody–drug conjugate (AXL-107-MMAE)	[80]
	BRAF, MEK	Dabrafenib, trametinib	PDX	RXR signaling	RXR (HX531)	[48]
Colon cancer	BRAF	Vemurafenib	VACO432, SNU- C5, HT29, KM20, WiDr; xenografts	EGFR-mediated feedback activation	EGFR (cetuximab, gefitinib, or erlotinib)	[95]
Colorectal cancer	BRAF	Vemurafenib	HT-29 and WiDr; xenografts	EGFR-mediated feedback activation	EGFR (erlotinib)	[96]
BCC	Hedgehog (Smoothened)	Vismodegib	Xenograft	Wnt signaling	Wnt (LGK-974)	[47]
Glioblastoma	RTK	Dasatinib	GSC6 and GSC8	Notch signaling	KDM (GSKJ4)	[45]
Various cancers	KRAS	ARS-1620, AMG 510	KRAS G12C cell lines, xenograft	RAS pathway feedback reactivation	SHP2 (SHP099)	[97]
**Metabolic reprogramming**						
NSCLC	EGFR	Erlotinib	PC9	ALDH upregulation to maintain ROS levels	ALDH (disulfiram)	[43]
Melanoma	BRAF	Vemurafenib	Primary cells	Bioenergetic metabolism (mitochondrial respiratory chain)	Mitochondrial enzymes (oligomycin and Bz-423, rotenone and phenformin, antimycin A, and oligomycin)	[60]
	BRAF, MEK	Vemurafenib, dabrafenib, trametinib	A375	Lipid hydroperoxidase (GPX4) pathway	GPX4 (RSL3)	[40]
Gastric	MET	Crizotinib	GTL-16, MKN-45	ALDH upregulation to maintain ROS levels	ALDH (disulfiram)	[43]
Breast	HER2	Lapatinib	BT474	Lipid hydroperoxidase (GPX4) pathway	GPX4 (RSL3)	[40]
**Interactions with the tumor microenvironment**						
Lung	EGFR	CL-387785	Cancer tissue-originated spheroids (CTOS)	MIG6/ERRFI1/RALT/Gene33 induction by hypoxia	PI3K (LY294002), MEK (trametinib)	[98]
Melanoma	BRAF	Vemurafenib	928MEL and 624MEL	Stromal secretion of HGF	MET (GDC-0712)	[16]
			SK-MEL-5, SK-MEL-28, G-361	Stromal cell secretion of HGF	MAPK (PD184352), PI3K–AKT (crizotinib, MK-2206)	[99]
			5555 and 4434	Activation of MAFs	FAK (PF573228, PF562271, FAKi14)	[100]

ALDH—aldehyde dehydrogenase; ALK—anaplastic lymphoma kinase; AURKA—aurora kinase A; AXL—Axl receptor tyrosine kinase; BCC—basal cell carcinoma; CDK—cyclin-dependent kinase; EZH2/PRC2—enhancer of zeste 2 polycomb repressive complex 2 subunit; EGFR—epidermal growth factor receptor; ERK—extracellular signal-regulated kinase; FAK—focal adhesion kinase; FGFR—fibroblast growth factor receptor; GPX4—glutathione peroxidase 4; HDAC—histone deacetylase; HER2—human epidermal growth factor receptor 2; HGF—hepatocyte growth factor; JAK—janus kinase; KDM5/6—lysine-specific demethylase 5/6; KRAS—Kirsten rat sarcoma; LINE—long interspersed nuclear element; MAFs—melanoma-associated fibroblasts; MEK; MAPK—mitogen-activated protein kinase; MET—tyrosine-protein kinase Met or hepatocyte growth factor receptor; NSCLC—non-small-cell lung carcinoma; PDCL—patient-derived cell lines; PDX—patient-derived xenograft; PI3K—phosphoinositide 3 kinases; RTK—receptor tyrosine kinase; SAC—spindle assembly checkpoint; SHP2—Src homology-2 domain-containing protein tyrosine phosphatase-2.

In addition to driving cell states or lineages that may be less reliant on oncogenic signals and more inherently drug-tolerant, epigenetic changes associated with persistence may also serve to protect cells from aberrant expression of repetitive or transposable elements, such as long interspersed nuclear elements (LINEs), short interspersed nuclear elements (SINEs, including Alu), and endogenous retroviruses (ERVs). These elements are typically epigenetically repressed, but can become activated in cancers, in part due to decreases in DNA methylation [101]. Interestingly, targeted therapies such as EGFR inhibitors and CDK4/6 inhibitors can induce a strong reactivation of repeated elements in cancer cells, which results in cell death [24,102]. In bulk populations of EGFR-mutant NSCLC cells, RNA sequencing revealed a drug-induced increase in expression of LINE-1 elements and IFN response/antiviral response genes after TKI treatment [24]. However, in DTPs H3K9 methylation accumulated at LINE-1 elements, preventing the increase in expression upon drug treatment, thus promoting cell survival. Treatment with HDAC inhibitors disrupted H3K9 methylation-dependent heterochromatin formation, thereby derepressing LINE-1 elements in DTPs and re-inducing cell death. Thus, suppression of repetitive elements by a repressive chromatin environment enforced by epigenetic remodeling may provide another mechanism by which persister cells can escape the toxic consequences of therapy.

### 3.2. Activation of Bypass and Alternative Signaling Pathways

Activation of bypass signaling pathways is a common mechanism of acquired drug resistance to targeted therapies in cancer, and therapeutic strategies that combine primary oncogene-targeted drugs with agents that block the emergent resistance-inducing bypass are being tested in the clinic in resistant patients (reviewed in [10]). For example, the discovery of MET amplification in a subset of resistant EGFR-mutant lung cancers [14] has now led to promising activity of combination EGFR and MET TKIs [103] in EGFR-mutant lung cancer patients with acquired resistance due to MET amplification. By analogy, activation of receptor tyrosine kinase bypass signaling pathways by exogenous growth factors can decrease the dependence of cancer cells on the targeted oncogene and facilitate drug tolerance [16]. Similarly, downregulation of negative feedback loops can lead to reactivation of oncogenic signaling that contributes to drug tolerance, a phenomenon often referred to as “adaptive resistance”. In both of these cases, reactivation of the same or very similar signaling pathways phenocopies the original oncogene. Adaptive resistance is especially important in BRAF and KRAS mutant cancers, that demonstrate reactivation of the MAPK signaling pathway. Multiple studies have demonstrated that in BRAF V600E mutant cancers, BRAF inhibition can lead to rapid EGFR-mediated feedback activation [95,96,104]. In BRAF mutant colorectal cancers, EGFR-mediated adaptive resistance severely limits the response to single-agent BRAF inhibition as well as combined BRAF and MEK inhibition; however, increased response rates to the triple combination of BRAF (dabrafenib), EGFR (panitumumab), and MEK inhibition (trametinib) have been observed in the clinic [105]. KRAS mutant cancers, particularly those harboring G12C activation, also appear to be particularly prone to adaptive resistance driven by receptor kinase signaling in response to KRAS G12C inhibitors or MEK inhibitors. Ryan et al. observed rapid adaptive RAS pathway feedback reactivation following treatment by KRAS G12C inhibitors ARS-1620 and AMG 510 [97]. This RAS pathway feedback reactivation was observed in the majority of KRAS G12C models and was driven by RTK-mediated activation of wild-type RAS isoforms and was refractory to inhibition by G12C-specific inhibitors. Co-treatment with SHP2 and KRAS G12C inhibitors abrogated feedback reactivation and led to sustained MAPK pathway suppression, resulting in improved efficacy in vitro and in vivo. Alternatively, a study by Xue et al. provided evidence that feedback activation of RTKs in response to G12C inhibitors can reactivate MAPK signaling in a G12C-dependent manner by shifting newly synthesized KRAS G12C protein into the active GTP-bound state that is not inhibited by the currently available G12C inhibitors that target the GDP-bound inactive state [106]. These findings echo prior studies showing that inhibition of RTK-driven feedback reactivation with SHP2 inhibitors can improve the response to MEK inhibitors [107,108,109].

Additionally, there is accumulating evidence that alternative signaling pathways involved in lineage and developmental pathways, innate immunity, and stress signaling play a crucial role in the survival of drug-tolerant cells. Several studies have shown that some drug-tolerant and -resistant states are characterized by increased expression of the AXL receptor tyrosine kinase, often in association with epithelial-to-mesenchymal transition (EMT). In the absence of the EGFR T790M mutation or MET activation, Zhang et al. found increased AXL activation and evidence for EMT in several in vitro and in vivo EGFR-mutant NSCLC models with acquired resistance to erlotinib [87]. Inhibition of AXL by genetic or pharmacological means restored erlotinib sensitivity in these tumor models. In EGFR-mutant lung cancers obtained from patients who had developed resistance to TKIs, increased expression of AXL and, in some cases, its ligand GAS6 was observed. In a more recent study, activated AXL was shown to associate with EGFR and HER3 in maintaining cell survival and inducing the emergence of EGFR-mutant NSCLC cells tolerant to osimertinib [92]. Early AXL inhibition reduced the viability of cells overexpressing AXL upon treatment with osimertinib. AXL was also highly expressed in a subset of EGFR-mutant NSCLC patients, and baseline high expression was associated with a lower response rate to subsequent EGFR inhibitor therapy, suggesting that a subset of cells with an AXL high drug-tolerant phenotype may pre-exist in EGFR TKI therapy. In melanoma cells, targeted therapy induced AXL expression, and co-treatment of AXL-expressing cells with an antibody–drug conjugate and MAPK inhibitors synergistically prevented tumor growth in melanoma patient-derived xenografts [80]. In addition to AXL, drug-tolerant mesenchymal DTPs may also rely on other receptor tyrosine kinases for survival. Raoof et al. showed that fibroblast growth factor receptor 3 (FGFR3) facilitates survival of mesenchymal EGFR-mutant drug-tolerant cells [93]. In these models, EGFR inhibitor therapy led to upregulation of both FGFR3 as well as several FGF family ligands. Long-term survival and expansion of drug-tolerant cells in vitro were inhibited by combining EGFR and FGFR inhibitors and prevented in vivo tumor relapse in xenograft models. Interestingly, in patient-derived models of EMT-driven acquired resistance, FGFR1, not FGFR3, was identified to drive resistance, suggesting a context-specific but convergent role for FGFR signaling in promoting EMT-associated drug tolerance/resistance. Together, these results suggest that up-front dual targeting of EGFR and FGFR or AXL might represent a strategy for preventing the emergence of mesenchymal drug-tolerant cells, analogous to the paradigm of using drug combinations to target bypass signaling pathways in the acquired resistance setting.

Several signaling pathways related to cell lineage, such as the Wnt/β-catenin pathway, RXR, and Notch signaling, have also been shown to be activated in tumor cells surviving drug treatment. The Wnt/β-catenin pathway is important in the development of epithelial states including alveolar type-2 (AT2) lung stem cells [110] that can serve as progenitor cells for lung adenocarcinomas [111]. In EGFR-mutant NSCLC models, Arasada et al. showed that EGFR inhibition resulted in the activation of β-catenin signaling in a Notch3-dependent manner [88]. This leads to the survival of a subpopulation of cells called “adaptive persisters”. In this case, a physical association between Notch3 and β-catenin resulted in increased stability and activation of β-catenin. Furthermore, the combination of EGFR-TKI and a β-catenin inhibitor suppressed the development of these adaptive persisters and improved overall survival of mouse tumor xenograft models, suggesting that combined EGFR and β-catenin inhibition might be a promising strategy for EGFR-mutant lung cancer. Sánchez-Danés et al. found that a slow-cycling LGR5+ tumor population promotes basal cell carcinoma (BCC) relapse after treatment with the Hedgehog (Hh) pathway Smoothened inhibitor vismodegib in both mouse and human BCC [47]. This persisting, slow-cycling LGR5-expressing population was characterized by active Wnt signaling, and combined Wnt and Hh inhibition resulted in tumor regression. A more recent study by Liu et al. observed increased Wnt/β-catenin signaling along with increased expression of the stem cell markers Oct4 and Nanog in gefitinib-resistant EGFR-mutant NSCLC cells and showed that over-expression of Oct4 and Nanog could stimulate Wnt/β-catenin signaling [112]. Other lineage drivers play a role in the context of different cell states. Applying single-cell RNA sequencing to BRAF-mutant patient-derived xenograft tumors treated with combination of RAF and ME inhibitors, Rambow et al. identified distinct drug-tolerant transcriptional states, including a neural crest stem cell-like (NCSC) transcriptional program largely driven by the nuclear receptor RXR [48]. Using the selective antagonist HX531 to target RXR signaling, a lower proportion of NCSC-like cells emerged upon MAPK inhibitor treatment, thus rendering melanomas more sensitive to treatment [48]. The Notch signaling pathway, one of the most commonly activated signaling pathways in cancer, is also implicated in the survival of persister cells. Liau et al. reported that glioblastoma persister cells depend on Notch signaling upon treatment with the multi-kinase inhibitor dasatinib [45]. On the other hand, Eberl et al. reported that Notch-activated suprabasal tumor cells in basal cell carcinoma (BCC) were more likely to undergo apoptosis in response to vismodegib treatment than basal cells with lower levels of Notch activation, indicating that low Notch levels may protect tumor cells from therapy [113]. These examples highlight the complexity and context-specific role of Notch signaling in drug tolerance and resistance.

Activation of inflammatory or innate immune signaling in cancer, such as IL-6/JAK/STAT3 axis, NF-κB, and interferon signaling, can promote tumor cell survival. Studies from the Sordella and Settleman groups have implicated a role of IL-6 in promoting drug tolerance in EGFR-mutant NSCLC models. By studying erlotinib-resistant EGFR models that had undergone EMT, Yao et al. identified that TGF-β induced expression of IL-6 and subsequent STAT3 activation could decrease erlotinib sensitivity [89]. Additionally, they identified that cells with stem-like properties and increased expression of TFG-β and IL-6 were present in cell lines and tumors even before therapy, consistent with pre-existence of persister-like cell states. Similarly, Lee et al., found that many oncogene-addicted cancer cells (including EGFR, HER2, ALK, MET, KRAS) engage an IL-6 dependent feedback loop leading to STAT3 activation [67]. This STAT3 activation promotes cell survival and limits the overall drug response. These findings suggest that inhibiting STAT3 signaling may improve the response to a wide range of drugs that target oncogene addiction pathways. Other studies by the Bivona group have demonstrated that activation of NF-κB signaling can facilitate tumor cell survival and residual disease in EGFR-mutant lung cancers treated with EGFR inhibitors. Using an RNA interference screen, they identified that knockdown of FAS and NF-κB pathway components enhanced cell death in EGFR-mutant lung cancer cells treated with EGFR TKI erlotinib [114]. Conversely, overexpression of c-FLIP or IKK, or silencing of IKB, in order to activate NF-κB, protected EGFR-mutant lung cancer cells from EGFR TKI therapy. In erlotinib-sensitive and erlotinib-resistant EGFR-mutant lung cancer models, genetic or pharmacologic inhibition of NF-κB increased erlotinib-induced apoptosis [114]. In a follow-up study, they showed that NF-κB signaling is rapidly engaged upon initial EGFR inhibitor treatment leading to activation of an NF-κB-mediated transcriptional survival program [90]. In multiple NSCLC models, including a patient-derived xenograft, the direct NF-κB inhibitor PBS-1086 suppressed this adaptive survival program and increased the magnitude and length of initial EGFR inhibitor response. Finally, a recent study by Gong et al. reported that EGFR inhibition in lung cancer triggers an adaptive response by co-opting interferon antiviral signaling pathways [115]. Mechanistically, EGFR inhibition triggered a type I interferon response via activation of the RIG-I/TBK1/IRF3 axis, and inhibition of interferon signaling enhanced EGFR TKI sensitivity in EGFR-mutant NSCLC. Surprisingly, while EGFR inhibition was capable of inducing interferon responses in both EGFR-mutant and wild-type lung cancers, this was NF-κB-independent in EGFR-mutant models but NF-κB-dependent in EGFR wild-type models.

### 3.3. Suppression of Apoptosis

Suppression of apoptosis, one of the classic hallmarks of cancer, has also been linked to drug tolerance and may represent an important therapeutic target for overcoming drug tolerance. Many studies have established a central role of the pro-apoptotic BH3 protein BIM in mediating the apoptotic response to targeted therapies (reviewed in [116]). Song et al. observed that resistant mesenchymal EGFR-mutant lung cancers exhibit an apoptotic defect due to loss of BIM expression, leading to loss of a cell death despite effective suppression of oncogenic signaling by targeted therapy [117]. This relationship between EMT and loss of BIM was also observed in KRAS-mutant lung cancers and other cancer subtypes. The authors demonstrated that EMT transcription factor ZEB1 suppresses BIM expression by binding directly to the BIM promoter and repressing transcription. Treatment with navitoclax (or ABT-263, a BH3 mimetic that inhibits the anti-apoptotic factors BCL-xL and BCL-2) to enhance “free” cellular BIM levels or derepression of BIM expression by depletion of ZEB1 both led to resensitization of mesenchymal EGFR-mutant cancers to targeted therapy. Similarly, resistant clones that evolved from drug-tolerant cells and retained a partial mesenchymal phenotype showed decreased apoptotic response to third-generation EGFR inhibitors, and treatment with navitoclax restored sensitivity [25]. Suppression of other pro-apoptotic proteins has also been shown to play a role in drug tolerance. Kurppa et al. observed that combined EGFR/MEK inhibition of EGFR-mutant NSCLC model cells selected a population of dormant drug-tolerant cells exhibiting senescence-like features characterized by high YAP/TEAD activity [56]. The EMT transcription factor SLUG cooperated with YAP/TEAD to blunt drug-induced upregulation of the pro-apoptotic BH3 protein BMF, thereby reducing drug-induced apoptosis. Drug-induced BMF expression and apoptosis were restored by pharmacological inhibition of YAP/TEAD, leading to a reduction in the survival of dormant cells.

Mechanisms associated with drug tolerance, such as EMT, may also indirectly suppress apoptosis. Nilsson et al. identified activation of a YAP/FOXM1 axis in EMT EGFR TKI-resistant models [91]. This was associated with dysregulation of spindle assembly checkpoint (SAC) proteins, including polo-like kinase 1, aurora kinases, and kinesin spindle protein, and rendered cells vulnerable to SAC inhibitors. An independent study by Shah et al. found that activation of aurora kinase A (AURKA) in TKI-treated drug-tolerant EGFR-mutant cells suppressed drug-induced apoptosis by increasing BIM phosphorylation and subsequent degradation [94]. Co-treatment with AURKA and EGFR inhibitors led to a synergistic response in vitro and in vivo, providing the rationale for the clinical investigation of this drug combination that is currently ongoing.

### 3.4. Metabolic Reprogramming

Metabolic dysregulation is a central hallmark of cancer, and increasing evidence suggests that cancer cells utilize metabolic reprogramming to enable survival in the face of oncogenic signaling blockade. Roesch and colleagues found that melanoma cell lines treated with diverse anticancer drugs, such as cisplatin, bortezomib, and the BRAF inhibitor vemurafenib, were enriched for resistant JARID1B^high^ melanoma cells with increased expression of mitochondrial proteins involved in bioenergetic metabolism [60]. These included enzymes such as NADH dehydrogenase and ATP synthase, which regulate oxidative phosphorylation, and pharmacologic blockade of the mitochondrial respiratory chain with ATP synthase inhibitors or complex inhibitors decreased JARID1B expression and promoted long-term growth inhibition in vitro. Examination of melanoma tumors from mouse models treated with vemurafenib, as well as patients who had become resistant to BRAF inhibition, revealed an increase in JARID1B-expressing cells, suggesting that cells with enhanced ability to utilize oxidative phosphorylation may be better poised to persist during therapy. To accomplish this, cells may rewire metabolic pathways to supply metabolites necessary for mitochondrial respiration to continue. In a study by Lue et al., inhibition of PI3K-mTOR led to suppression of glycolysis and triggered autophagy in a renal carcinoma model [118]. The authors hypothesized that the mobilization of lipids fueled mitochondrial oxidative phosphorylation and demonstrated that inhibition of phospholipase A2 (PLA2) or deletion of the autophagy gene ATG5 suppressed oxidative phosphorylation and increased apoptosis.

One consequence of metabolic dysregulation in cancer cells in general, and persister cells in particular, is an increase in the production of reactive oxygen species (ROS). Drug-tolerant persisters thus depend on antioxidant pathways to mitigate oxidative stress. Raha et al. reported that a wide variety of DTP experimental models representing lung, breast, colon, and gastric cancers treated with targeted therapies rely on expression of aldehyde dehydrogenase (ALDH) to protect from the toxic effects of high levels of ROS in these cells on the drug-tolerant subpopulation [43]. Pharmacologic inhibition of ALDH activity resulted in accumulation of ROS to toxic levels, causing DNA damage and cell death within the drug-tolerant subpopulation, suggesting that oxidative stress pathways may be a targetable vulnerability of DTPs. This concept has been expanded by several studies showing that drug-tolerant cells with mesenchymal features are dependent on phospholipid glutathione peroxidase 4 (GPX4), a selenocysteine-containing enzyme that detoxifies lipid peroxides. Specifically, GPX4 plays a key role in preventing the iron-mediated reactions of peroxides that induce ferroptosis, a recently described form of non-apoptotic programmed cell death characterized by iron-dependent lipid peroxidation. Mesenchymal cells marked by high ZEB1 expression appear to be especially sensitive to GPX4 inhibition [119]. Drug-tolerant persister cells that have a disabled antioxidant program, characterized by lower levels of reduced glutathione (GSH) and NADPH levels as well as increased oxidative stress, are reliant on GPX4-mediated suppression of ferroptosis for survival, demonstrated by the fact that cell death induced by GPX4 inhibition could be reversed by treatment with lipophilic antioxidants ferrostatin-1 and liproxstatin-1, an iron chelator, but not caspase inhibition [40]. Interestingly, diverse therapy-resistant states characterized by high expression of ZEB1 exhibit this dependency on GPX4. This includes epithelial-derived carcinomas that had undergone EMT, TGF-β-mediated therapy resistance in melanoma, treatment-induced neuroendocrine transdifferentiation, sarcomas, and a persister state derived by chronic exposure to lapatinib. While the validation of this hypothesis awaits the development of selective GPX4 inhibitors with in vivo activity, the broad applicability in cell line models raises enthusiasm for the potential of this approach as a therapeutic strategy for preventing acquired drug resistance.

### 3.5. Interactions with the Tumor Microenvironment

The tumor microenvironment is a complex ecosystem comprised of multiple cell types that support the growth of tumor cells, facilitate tumor progression, and modulate therapeutic response to anti-cancer treatments [29,99,100,120,121]. While a full discussion of the tumor microenvironment, and in particular the role of the innate and adaptive immune system, is beyond the scope of this review, we highlight several features of the stromal microenvironment that can facilitate the survival of drug-tolerant cells by modulating tumor intrinsic survival pathways.

The potential role of cancer-associated fibroblasts (CAFs) in fostering resistance to therapy has long been appreciated, both through secretion of soluble factors and direct cell-to-cell contact. Through their ability to activate receptor tyrosine kinases and the same downstream signaling pathways as the targeted oncogene, many soluble growth factors have the ability to oppose the effects of a range of targeted therapies [16,99]. The prototypical soluble microenvironment factor, HGF, has been shown to be produced by CAFs and rescue cells in a number of contexts through activation of the MET signaling pathway. Wang and colleagues demonstrated that a subset of primary CAF cultures from EGFR-mutant NSCLCs express HGF and this is sufficient to decrease response to EGFR targeted therapy both in vitro and in vivo [122]. Similarly, HGF activation of the MET signaling pathway can decrease the response of BRAF-mutant melanomas to BRAF inhibition. By screening cancer-fibroblast co-cultures with a panel of cytotoxic and targeted agents, Straussman et al. identified that HGF-secreting fibroblasts rescued BRAF mutant melanoma cells from the BRAF inhibitor PLX4720 [99]. Using either an HGF-blocking antibody or the MET small molecule inhibitor crizotinib to target the HGF–MET axis suppressed fibroblast-mediated resistance. Examination of BRAF-mutant melanoma patients treated with BRAF and/or MEK inhibitors revealed that expression of HGF in tumor-associated stromal cells inversely correlated with response to therapy, and perhaps most importantly, on-treatment biopsies taken 2 weeks after initiation of therapy showed an increase in stromal HGF expression in a subset of patients, suggesting that stromal HGF may contribute to tumor cell persistence early in the course of therapy. Beyond secretion of growth factors, CAFs can remodel local stromal microenvironment niches to promote the survival of cancer cells during drug treatment. In preclinical melanoma models, Hirata and colleagues showed that tumor cells present in areas of high stromal density display rapid reactivation of MAPK signaling after BRAF inhibitor treatment. Experiments using tumor-CAF co-culture systems revealed that BRAF inhibition led to paradoxical activation of CAFs and altered matrix production remodeling. The increase in extracellular matrix stiffness activated integrin, FAK, and Src signaling in melanoma cells that ultimately enabled them to become drug tolerant by adaptive reactivation of MAPK signaling [100]. Inhibition of this signaling loop increased response to BRAF inhibitor treatment, suggesting that strategies that target stromal–tumor interactions may have therapeutic potential.

Finally, general features of the tumor microenvironment such as the density and distribution of the vasculature can influence the fitness of tumor cells and subsequent emergence of drug-resistant clones through effects on nutrient and oxygen levels [60]. In melanoma cells, a dynamic transition between a proliferative state and a drug-tolerant slow-cycling state was observed in response to varying oxygen levels [60]. In low oxygen conditions, cells activate hypoxia-inducible factors and upregulate the expression of key dormancy genes such as MIG6, resulting in a dormant and drug-resistant state [98]. In hepatocellular carcinoma, long-term treatment with sorafenib deprives tumors of oxygen over time, which can contribute to the proliferation of cancer cells that can tolerate low oxygen levels, which in part may explain the clinical observation that increased expression of HIF-1α and HIF-2α in HCC patients are markers of poor prognosis [123].

### 3.6. Adaptive Mutagenesis

One mechanism that bacteria use to improve survival upon antibiotic exposure is to transiently increase their mutation rates (adaptive mutability) to generate phenotypic diversity that increases the probability of survival of sub-clones within the bulk population. Emerging evidence suggests that cancer cells may adopt similar behavior. Russo et al. examined human colorectal cancer (CRC) cells treated with EGFR antibodies or BRAF inhibitors and found that that drug-tolerant persister cells downregulate mismatch repair (MMR) and homologous recombination (HR) DNA repair genes, whereas error-prone polymerases are upregulated [124]. During treatment, MMR proteins were also downregulated in tumor specimens and patient-derived xenografts. While it remains to be clarified whether this directly contributes to the drug-tolerant phenotype per se, or generates population heterogeneity with increased probability that some clones will have a fitness advantage, these results suggest parallels between tumor cells and bacteria in the ability to evade therapeutic pressure by enhancing mutability.

## 4. Persisters in the Clinic

The most relevant clinical analogy to the experimental drug-tolerant persister state is minimal residual disease (MRD) in patients with advanced or metastatic cancer receiving continuous treatment with systemic targeted therapies (Figure 3C). While many of these patients experience dramatic initial tumor regression, these responses are incomplete and typically followed by a prolonged period in which the residual tumor lesions appear dormant on radiological imaging. While it is atypical to obtain tissue samples of these MRD lesions as part of routine clinical care, the observation that disease relapses typically occur at these sites (sometimes after years of dormancy) provides evidence that viable cancer cells persist at MRD sites. Although residual disease could be secondary to several mechanisms (including pharmacokinetic problems that prevent full target inhibition), the presence of drug-tolerant persister cells with phenotypes discussed above seems likely [125]. Currently, clinical trial designs that incorporate on-treatment biopsies provide the best opportunities to directly study persisters in the clinical setting (Figure 3C). A therapy-induced persister state may also have clinical relevance to dormant cancer cells that can drive long-term relapse in patients who have received definitive therapy and are considered clinically disease-free. For example, in treated localized breast cancer, late relapse can occur locally or in distant organs years or even decades after resection of the primary tumor [126,127]. This scenario of clinical cancer dormancy, which occurs in the absence of continued therapy, nevertheless reinforces the notion of non-linear tumor growth driven by slow-growing cancer cells that may possess features similar to drug-tolerant persisters. At present, our understanding of the nature and role of drug-tolerant persister and dormant tumor cells in clinical minimal residual disease and subsequent disease relapse is limited by the difficulty of accessing access tumor tissue at this time point for study. Moreover, molecular signature(s) in clinically relapsed tumors that may indicate whether the resistant cells passed through a persister state have yet to be defined. Thus, the true extent of clinical disease relapse that results from the evolution of drug-tolerant persister cells versus the out-growth of pre-existing resistant clones remains unknown.

Attempts have been made to apply mathematical modeling to estimate the contribution of persister cells to clinical drug response and resistance. Starting from 3D volume estimates of serial computerized tomography (CT) scans, Grassberger et al. used mathematical models to estimate the size of resistant and persister populations in EGFR-mutant lung cancer patients during treatment with EGFR inhibitors [128]. The calculated growth trajectories revealed that neither a pure persister evolution nor a pure pre-existing model of resistance was sufficient to explain the clinical response and subsequent development of resistance that was observed in this cohort of patients, all of which developed T790M-mediated resistance. Instead, a model combining pre-existing resistant and persister cells matched the observed tumor volume trajectories, in line with clinical studies that have shown that T790M-resistant tumors are comprised of a heterogeneous population of T790M-positive and -negative sub-clones [129].

The advent of single-cell RNA sequencing (scRNA-seq) has enabled the ability deconvolute transcriptional phenotypes within heterogeneous cell populations in clinical tumor samples, thus providing a means for characterizing drug-tolerant persister cells that may exist in small numbers in minimal residual disease tumors. In a recent study of advanced-stage NSCLC patients treated with targeted therapies, Maynard and colleagues performed scRNA-seq on biopsies collected from patients before initiating therapy, at residual disease (any period during targeted therapy when clinical imaging showed the tumor was regressing or stable), and at disease progression [130]. Tumor cells from on-treatment residual disease biopsies showed increased expression of genes corresponding to an alveolar-regenerative cell signature, suggesting that drug-tolerant persister cells adopt a cell state reminiscent of alveolar stem-like progenitors with increased WNT/B-catenin signaling that enables them to survive therapy. The authors also performed scRNA-seq on the cells from the tumor microenvironment and found that the immune composition at residual disease featured decreased numbers of macrophages and increased active T-lymphocytes compared with treatment-naive and -resistant tumors, suggesting that residual disease state may be characterized by an immunostimulatory microenvironment.

## 5. Conclusions

In summary, cells have developed similar redundant strategies where the role of survival is allocated to a small dormant subpopulation of cells within a more rapidly proliferating population, whether microbial or human in nature. In cancer, the prospect that these drug-tolerant persister cells serve as a reservoir for subsequent evolution of diverse stable drug resistance mechanisms makes them attractive targets for new therapies. However, the success of strategies that target persister cells will depend heavily on the extent of our knowledge of the mechanisms driving drug tolerance in heterogenous subpopulation of cells in specific clinical contexts. Thus, an important objective of future work should be to continue prioritizing efforts to obtain on-treatment biopsies from patients at the point of minimal residual disease. These studies will be essential to understand the biology of persistence and determine how faithfully the clinical reality is modeled by the experimental models on which the majority of our current understanding of drug tolerance mechanisms and tumor cell persistence is based. Moreover, these efforts will also be important for dissecting the role of the tumor microenvironment, which cannot be addressed well by in vitro models or in vivo studies using immunocompromised mice. The continued evolution of single-cell sequencing methods, as well as tissue-based profiling, such as spatial transcriptomics, highly multiplexed immune fluorescence, and mass spectrometry-based approaches, will allow for a deeper characterization of drug-tolerant persisters cells as well as the surrounding tumor microenvironment in clinical samples. Although the transient nature and low abundance of these cells have made them historically challenging to study, our developing knowledge of the role they play in the evolution of drug resistance and tumor progression will undoubtedly continue to motivate efforts to target them.
